# Short-term outcome of fragility fractures of the pelvis in the elderly treated with screw osteosynthesis and external fixator

**DOI:** 10.1007/s00068-021-01780-3

**Published:** 2021-09-21

**Authors:** Konrad Schuetze, Alexander Eickhoff, Christoph Dehner, Alexander Blidon, Florian Gebhard, Peter Hinnerk Richter

**Affiliations:** Department of Trauma-, Hand-, and Reconstructive Surgery, Ulm University, Albert-Einstein-Allee 23, 89081 Ulm, Germany

**Keywords:** Pelvic fracture, Sacral fracture, Fragility fracture, Navigation, Geriatric trauma

## Abstract

**Background:**

The treatment of fragility fractures of the pelvis is rising challenge for orthopedic trauma surgeons. Operative treatment should allow immediate full weight bearing and early mobilisation but should also be as minimal invasive as possible. Sacroiliac (SI) or transsacral transiliac screws (TSTI) alone or depending on the fracture in combination with an external fixator meets both of these criteria.

**Material and methods:**

The outcome of 121 operatively treated patients with fragility fractures of the pelvis were evaluated in this retrospective study. Depending on the type of fracture the patients were treated with navigated SI screw or TSTI screw alone or in combination with an external fixator. All patients were operated in supine position in a hybrid-OR, which consists of a fixed robotic 3D flatpanel detector (Artis zeego, Siemens Healthineers, Germany) and a navigation system (BrainLab Curve, BrainLab, Germany).

**Results:**

37 patients were treated with either one or two SI screws and 57 with one TSTI screw. An additional external fixator was combined with SI screws in 17 patients and with TSTI screws in 10 patients. The preoperative pain score was significantly higher compared to the postoperative score (5.1 ± 2.5 vs 2.2 ± 1.9, *p* < 0.05). Follow-up at 6 month was possible for 106 patients which showed screw loosening in 16.3% of the SI Screws (*n* = 49) compared to only 5.2% of TSTI screws (*n* = 57). No screw loosening was seen in the combination of TSTI-screw and external fixator (*n* = 10). There were two septic and three aseptic pin loosenings of the external fixator. Overall only one patient needed revision surgery due to screw loosening and local irritation. Overall 75.2% (*n* = 91) of the patients could be released in their home or in a rehabilitation unit and only 14% (*n* = 17) were released to a nursing home due to immobility despite the operation. Non-surgical complications rate was 21.5%.

**Conclusion:**

SI or TSTI screws with possible combination with an external fixator show early pain relief and allows most of the patients to keep their former level of independence. With an also low surgical complication rate, it proved to be a safe and reliable treatment for fragility fractures of the pelvis. Due the effective pain relief and the minimal invasive approach, early mobilisation is possible and might prevent typical non-surgical complications which are very common during conservative treatment.

## Background/Introduction

Osteoporotic fractures of the pelvic ring in elderly patients are a rising challenge for orthopaedic trauma surgeons. Due to the fast growth of the elderly population these insufficiency fractures show a steady increase over the last years [1]. Patients mostly present with lower back pain and fractures are often missed in conventional radiographs [2]. Computed tomography (CT) images are mandatory to diagnose fragility fractures of the sacrum, but some fractures can only be seen in magnetic resonance imaging (MRI). Rommens and Hofmann classified insufficiency fractures of the pelvic ring in great detail [3] and the established treatment algorithm in this study for the different types of pelvic ring fractures followed the recommendations based on this classification [4]. The fragility fractures of the pelvis (FFP) Type IA and IB can be treated conservatively, but a fracture of the sacrum must be ruled out with CT or MRI. While FFP2 fractures should only be treated operatively, if early mobilisation is not possible, FFP3 and FFP4 fractures should always be treated operatively. Still factors like the mobility before the fracture, comorbidities, pain levels and the mental state should be considered carefully while deciding between conservative and operative treatment and when planning the type of operative treatment.

There is evidence that conservative treatment of FFP2-4 fractures leads to prolonged hospitalization, increased mortality and a major loss of autonomy [5–7]. In comparison, a few studies found lower pain scores and a better overall outcome for operative treatment compared with conservative treatment [8, 9]. There is an ongoing discussion about the best fixation techniques, which are ranging from lumbopelvic fixation to sacral bars or long transsacral transiliac (TSTI) screws [4]. These fixation techniques are well established in young non osteoporotic patients. Current literature shows also promising results in the treatment of fragility fractures of the pelvis but only in small study populations. Mehling et al. showed in 11 included patients that sacral-bars can improve clinical outcome without adverse surgical events [10]. Schmitz et al. showed for the cement-augmented screw-rod system good postoperative mobility in 10 out of 15 patients but had surgical complications in 5 out 15 patients [11]. For lumbopelvic fixation, there is only one case report reporting good clinical outcome [12] and compared to the other techniques it is more invasive. Percutaneous screw stabilization is often used for osteoporotic fractures, because they can be implanted minimally invasive and ensure early mobilisation at low perioperative risk. Main concern is malposition of the screws with potential damage to neurovascular structures and fixation in the osteoporotic bone. Malposition rates have been reported from 2 to 15% [13–15] with an incidence of neurological injuries between 0.5–7.7% [15]. To reduce malposition rates, intraoperative navigation is increasingly used for treatment of fragility fractures of the pelvis and shows lower rates of screw perforations and neurological injuries but might be associated with higher costs. To improve screw fixation, biomechanical studies showed better results for augmented sacroiliac (SI) screws and TSTI screw fixation [16, 17] compared to non-augmented short screws. Still, augmentation of the screws bears the risk of cement leakage [18] and TSTI a higher risk of malposition and neurological damage. Stabilization of the anterior pelvic ring can be achieved with minimal invasive screws, external or internal fixator or plate osteosynthesis. Minimal invasive screw osteosynthesis bears the risk of misplacement and loosening [19], while open reduction and plate fixation shows high rates of screw loosening and is not possible minimal invasive [20]. In comparison stabilization with an external fixator is minimal invasive and simple and can lead to considerable pain relief and improved mobility. Still the risk of pin loosening and pin track infection remains and there is a considerable risk of self-harm or manipulation [21]. A subcutaneous screw rod system (INFIX) shows good clinical and radiological results with a low complication rate in patients below 65 years [22] but is lacking studies in geriatric patients with fragility fractures. Furthermore, it is technical demanding and some studies report lateral femoral cutaneous nerve irritation up to 48% [23].

Therefore, the authors of this study used intraoperative 3D navigation and SI or TSTI screws for fragility fractures. An additional supraacetabular external fixator can be required depending on the fracture morphology. SI and TSTI screws were implanted in a hybrid OR facilitating advanced intraoperative imaging and 3D navigation. Aim of the study was to evaluate the outcome of operatively treated patients with fragility fractures of the pelvis regarding to their pre- and postoperative pain score, discharge disposition as well as surgical-and non-surgical complications.

## Methods

**Fig. 1:**
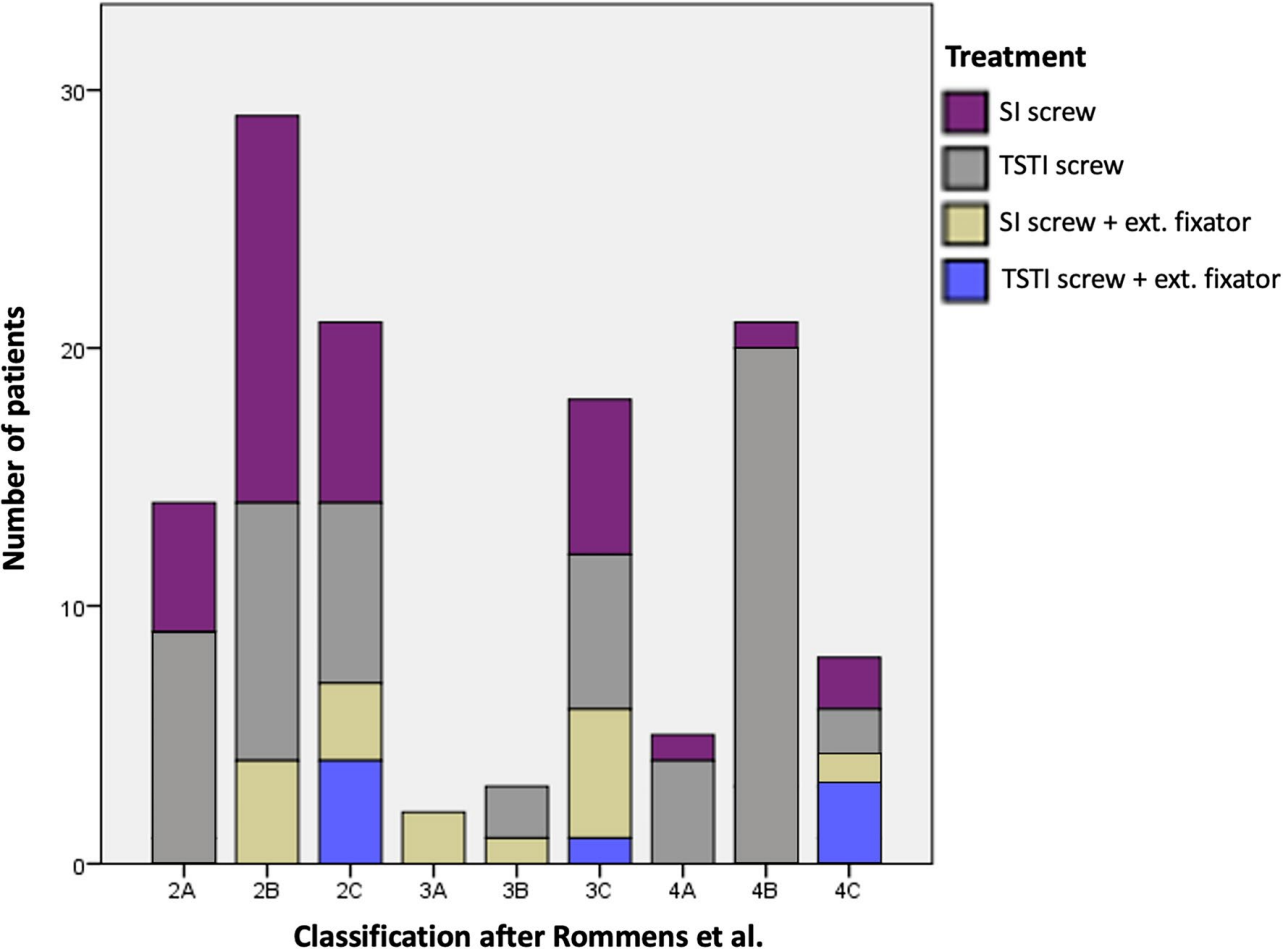
Number of patients classified according to Rommens and Hofmann with type treatment

Institutional and prior ethical committee approval for the use of data was obtained. Between January 2014 and December 2019, 121 patients with osteoporotic fractures of the pelvic ring, treated with percutaneous SI or TSTI screws, were included in the study. All patients sustaining ground-level falls or with no distinct history of trauma that were treated operatively for a fragility fracture of the pelvis were included. All fracture were classified according to the classification of fragility fractures of the pelvic ring of Rommens and Hofmann [3]. Patients with FFP2 fractures received conservative treatment in the beginning with physiotherapy, pain medication and thromboembolism prophylaxis. Following our clinical therapy regime, only patients, who could not be mobilized within 3 days despite physiotherapy and pain management or with FFP3-4 fractures, were treated operatively. The treatment option was chosen individually for each patient based on physical status and fracture morphology mainly according to the recommendation of Wagner et al. [4]. Overall, 37 patients were treated with either 1 or 2 SI screws and 57 with TSTI screws. Due to SI screw loosening in undisplaced FFP2B fracture, the authors implanted an increasing number of TSTI screws even in FFP2A-C fractures during the study period. An additional external fixator was combined with SI screws in 17 patients and with TSTI screws in 10 patients. Fracture classification and type of treatment shows Fig. 1.

All patients were operated in supine position in a hybrid-OR, which consists of a fixed robotic 3D flat panel detector (Artis zeego, Siemens Healthineers, Germany). It is linked to an operating table (Trumpf, Germany) and a navigation system (BrainLab Curve, BrainLab, Germany). After a reference base was fixed to the iliac crest, an intraoperative 3D scan was performed and automatically sent to the navigation system. A 3.2-mm k-wire was inserted using a navigated drill guide according to the planned trajectory. With a second scan the position of the k-wire was examined and a 7.3-mm cannulated screw with a washer was implanted. A supraacetabular external fixator (ext. fixator) was implanted if the anterior pelvic ring was highly dislocated or if only SI-screws were implanted to increase overall stability. To prevent self-harm and manipulation the external fixator was not used in patients with dementia. Overall, the decision was made carefully and individually for each patient based on the mentioned factors. The external fixator was implanted following AO-principles with two pins which were connected with a rod-to-rod clamp and was removed after 6 weeks or if showing signs of loosening or infection. Full weight bearing was allowed and was only not possible in three patients with highly restricted preoperative mobility.

For this retrospective study, clinical records including patient charts, laboratory results and radiographic images were reviewed. Patient-related factors like age, gender, body mass index and ASA classification were recorded. Preoperative computed tomography images were used to classify the fractures applying the classification of fragility fractures of the pelvic ring [3]. Follow-up was standardized at 6 weeks and 6 months postoperatively with clinical examination and X-ray images.

The primary outcome measures were pre- and postoperative Visual Analog Scale pain scores and discharge disposition. The postoperative pain score was always recorded on the third postoperative day to avoid measuring pain that is mainly related to the operative procedure. Also pre- to postoperative hemoglobin difference and transfusion rate during hospital stay was recorded. The secondary outcome measures were mortality and surgical complications within 6 months. Also, non-surgical complications, time to surgery and length of hospital stay was evaluated. Loosening of the screws, deep wound infection, postoperative neurological symptoms and major screw-perforation were counted as surgical complications. As non-surgical complications deep vein thrombosis, cardiac infarction, stroke, pneumonia, urinary tract infection, acute renal failure was defined.

Data analysis was performed with IBM SPSS Statistics (V21.0) and Microsoft Excel (V16.3). Demographic characteristics are described as mean and standard deviation. For the primary outcome measures, logistic regression was performed considering all variables related to the pre- to postoperative difference on the visual analog scale. Also, for the secondary outcome measures logistic regression was performed considering all variables related to screw loosening.

## Results

### Patient population

For 121 patients, medical records were reviewed. Out of these 121patients, 32 were male with a mean age of 73.9 ± 9.7 years. Mean age of the included women (*n* = 89) was 78.4 ± 8.5 years. Average body mass index was 25.6 ± 4.4. 21 patients were preoperatively classified as ASA II, 80 as ASA III and 19 as ASA IV. Mean operating time was 53.8 ± 45.2 min. The mean pre- to postoperative Hb-difference was 0.5 ± 1.1 g/dl. 10 patients needed a transfusion during the hospital stay, all of them had an Hb below 8 g/dl at admission. Follow-up was possible in 116 patients after 6 weeks and 106 patients after 6 months.

### Fracture classification

All fractures were classified according to the classification of fragility fractures of the pelvic ring of Rommens and Hofmann [3]. Fracture classification and type of treatment for all patients are shown in Fig. 1.

### Pre- and postoperative pain

The preoperative visual analog scale pain score was significantly higher compared to the postoperative score (5.1 ± 2.5 vs 2.2 ± 1.9, *p* < 0.05). The significant average pain reduction was 3 grades on the visual analog scale (*p* < 0.05) on the third postoperative day. 118 patients were mobilized with full weight bearing postoperatively. The three remaining patients were only mobilized in a wheelchair due to preoperatively already restricted mobility. Logistic regression showed no significant influence for the factors BMI, weight bearing or fracture classification on the pain score.

### Hospital stay and discharge disposition

Mean time to surgery was 5.3 ± 1.5 days and mean hospital stay was 16.7 ± 10.2. 47 patients could be released directly home after the hospital stay, 53 were discharged into geriatric rehabilitation and 21 in a nursing home. 8 out of 21 patients released to a nursing home, already lived there before the fall. Out of the 53 patients released into geriatric rehabilitation 44 returned home after rehabilitation, 4 were discharged to a nursing home. For five patients, it was retrospectively not possible to evaluate where there were discharged after geriatric rehabilitation. Overall 75.2% (*n* = 91) of the patients could be released home but 14% (*n* = 17) of the patients could not return into their normal lives and needed to be admitted to a nursing home despite the operation and geriatric rehabilitation.

### Surgical complications

Surgical complications were low with no patients showing neurological symptoms postoperatively. 106 of 121 showed no perforation of the screws and the remaining 15 patients showed minor perforations with less than 4 mm. 2 deep tissue infections were recorded. Both cases were associated with an external fixator and needed no further treatment after removal in the first case after 3 weeks and in the second after 4 weeks. No revision surgery was necessary in both cases due to good mobility and low pain levels. The external fixator showed signs of aseptic loosening in three patients in the controls after 6 weeks before planned removal.

### Screw loosening

**Fig. 2:**
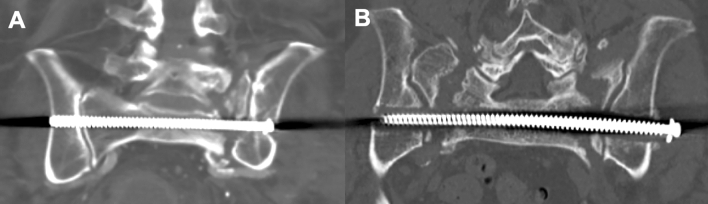
Pseudarthrosis after 6 months in CT-scan without loosening (**A**) and with minor loosening of a TSTI screw (**B**)

**Fig. 3:**
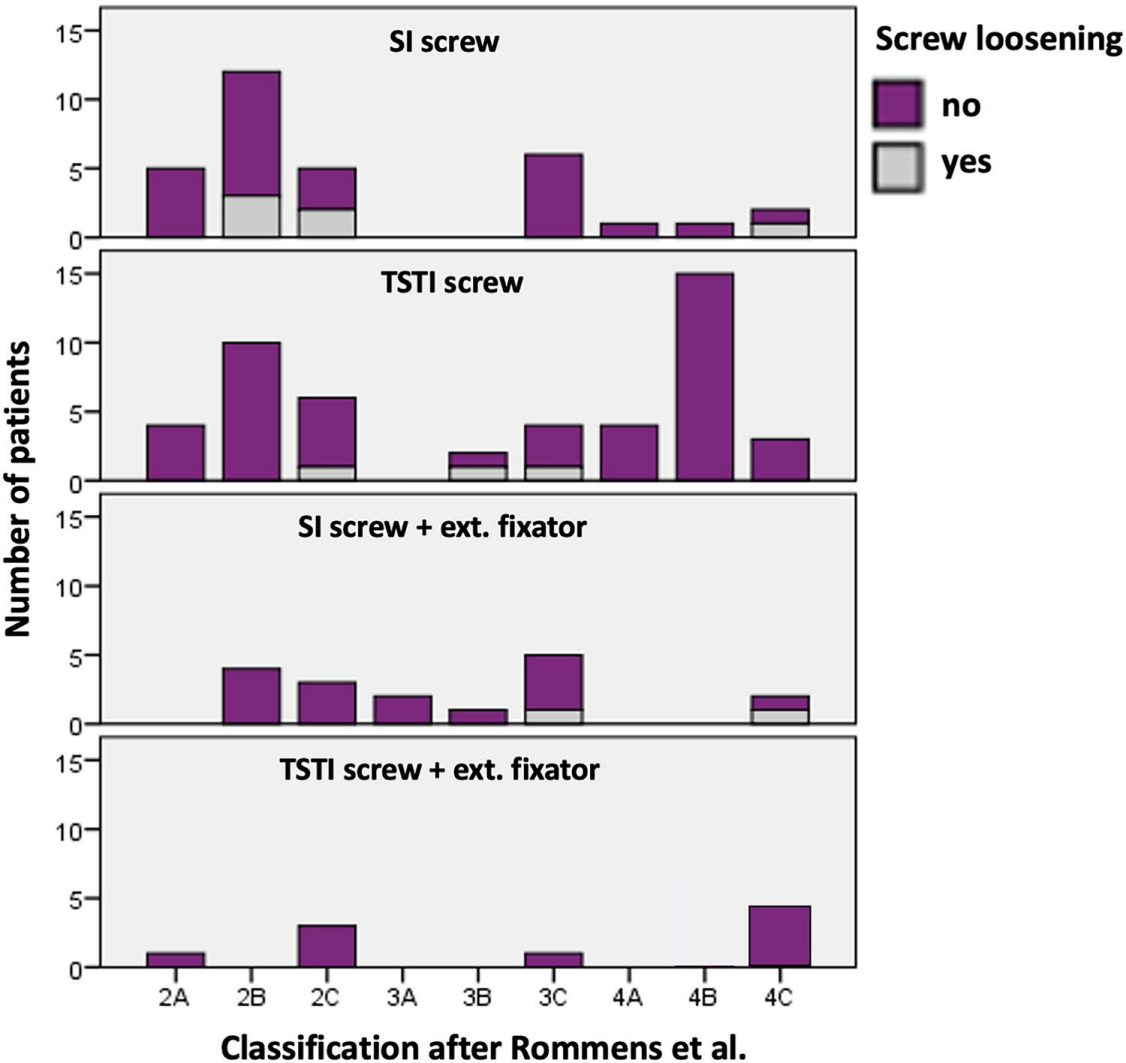
Number of cases with screw loosening sorted by fracture classification and type of treatment

Only 106 patients were followed up over at least 6 months. Screw loosening like shown in Fig. 2 was seen in 11 of these cases. Logistic regression showed no dependence to BMI, weight bearing, fracture classification, time to surgery or type of treatment. 8 out of 49 SI screws showed signs of screw loosening, while only 3 out of 57 TSTI screws loosened (16.3% vs. 5.2%). Still, the rate of screw loosening showed no significant difference between SI and TSTI screws (*p* = 0.270). Figure 3 shows screw loosening in dependence of fracture classification and chosen treatment option. Screw loosening was seen first after 6 weeks in all the cases. In ten cases, the patients did not complain about any increased pain due to loosening. In only one case the loosened screw had to be removed because of local pain.

### Non-surgical complications and mortality

21.5% of the patients had non-surgical complications. Most common complications were urinary tract infections in 18 patients. 4 patients were treated for pneumonia, two for pulmonary embolism, two for acute renal failure and one for myocardial infarction. Within 6 months, out of the 106 patients that could be followed up, 2 died. Mortality was low with 1.9%.

## Discussion

Treatment of fragility fractures of the pelvis remains a challenge for orthopaedic trauma surgeons. There is an ongoing discussion about the best timing of the operation and treatment option. Studies with long term outcome or large numbers of patients for the various treatment options like lumbopelvic fixation [12], sarcoplasty [24], sacral bars [10] or SI screws [25, 26] are rare. There is evidence that conservative treatment leads to high rates of mortality and a loss of social and physical independence and autonomy [6]. Thus, if early mobilisation is not possible, surgical treatment should be considered. The authors of this study use SI and TSTI screws, which were implanted percutaneously in supine position using a hybrid-OR and 3D-navigation. Depending on the anterior fracture type and dislocation, SI and TSTI screws were combined with a supraacetabular external fixator. This study shows that all types of fragility fractures can be treated successfully in supine position with this percutaneous procedure, without open reduction, long operating times or significant blood loss.

To verify the positive outcome, the visual analog pain scores were compared pre- and postoperatively and showed a significant average improvement of three grades. This is comparable to the study of Hopf et al. [25] and Pulley et al. [27], which also used SI and TSTI screws. Studies showing an even higher improvement, measured the pain score later than the third postoperative day [8, 9]. Furthermore, these results are comparable to the outcome after sacroplasty without the risk of cement leakage [24]. Only 14% of all patients in this study lost their independence and had to be referred to a nursing home despite operative treatment. Therefore, 75% of the patients could be discharged to their home directly or after geriatric rehabilitation. This is a strong improvement compared to the reported outcome of conservative treatment by Breuil et al. [5], with only 30% of the patients being able to live at home after a pelvic ring fracture. Maier et al. also showed a high loss of autonomy after conservative treatment compared to our results. Furthermore, compared to our study Maier et al. had 50% FFP1a and no FFP3-4 lesions [6]. Mortality rate was low compared to reported rates after conservative treatment [7, 28]. Also, non-surgical complications might have been prevented due to early mobilization. In our study, rate of non-surgical complications was 21.5%, while non-surgical complications were reported up to 58% after conservative treatment [6]. Overall, this underlines the importance of surgical treatment, if early mobilization is not possible.

In contrast to recommendations of Rommens and Hofmann, we treated all patients as minimalistic as possible and avoided any kind of open reduction. Even patients with FFP4C fractures were treated with TSTI screws and external fixator. Overall, only in cases with high grades of dislocation of the anterior pelvic ring fracture an external fixator was implanted in addition to SI or TSTI screws. Due to the minimal invasive approach blood loss and transfusion rate as well as operating time was low, still bearing the risk of non-union which might result in persisting pain and immobilization.

Due to the percutaneous approach and 3D navigation, surgical complications like screw perforations or loosening of the external fixator were low compared to current literature [13].

There were only two pin track infections, which needed no further treatment after removal of the external fixator. 3 cases showed aseptic loosening of the external fixator after 6 weeks. So overall complication rate of the external fixator was 18.5%. This is comparable to the surgical complications rate of anterior plate or screw fixation [19, 29] {Herteleer:2021ed} but is less invasive and if loosening occurs the external fixator is easily removed. As an alternative, a subcutaneous screw rod system (INFIX) showed good clinical results in younger patients [22], but also some rare but major complications [23].If the INFIX is a valuable option in geriatric patients has to be evaluated in further studies. Still, particularly in patients suffering from dementia it might prevent self-harm and manipulation. Compared to similar studies [7, 25], no revision surgery was needed because of screw malposition. Due to improved visibility and feasibility of 3D-navigation, SI and TSTI screws could be implanted with high accuracy in the hybrid-OR [29, 30]. Still there was screw loosening in 11 out of 121 cases, which needed revision surgery in 1 case due to local irritation and pain. Screw loosening was reported slightly higher in the study from Eckardt et al. in 9 out of 50 cases [26]. Unfortunately, both studies failed to find significant risk factors for screw loosening. Pulley et al. found no screw loosening of TSTI screws in sacral U-type fractures [27]. In line with these findings only 5.2% of the TSTI screws compared to 16.3% SI screws showed signs of loosening. The tendency for more overall stability for TSTI screws is in line with biomechanical studies showing increased stability for TSTI screws [16]. Therefore, the authors used an increasing number of TSTI screws even in FFP2A-C fractures during the study period. Heydemann et al. showed that stabilization of uninjured sacroiliac joints withs TSTI screws did not influence pain or functional outcome. So TSTI screws might improve overall stability without affecting the uninjured sacroiliac joint and can therefore be safely used in FFP2A-C fractures. In FFP3C and FFP4C cases screw loosening was only seen in cases with SI screws and external fixator, but not when TSTI screws and an external fixator was used. Our data suggest that in addition to a TSTI screw an external fixator might also prevent screw loosening especially in FFP3C and FFP4C fractures. The difference between these groups was not significant most likely due to the small number of cases and needs to be evaluated in further studies. In ten cases, the loosening was minor and no further surgical treatment was necessary. All of these patients had improved visual analog pain scores compared to preoperatively. Only one case showed major loosening with local irritation and pain, so the screw had to be removed.

TSTI screw can be safely implanted using 3D navigation even in a sacral dysmorphism [29]. In these cases, the S2 corridor has to be used frequently for screw placement. Therefore, TSTI screws became the standard treatment in the author’s institution for FFP2A–FFP4C in combination with a supraacetabular external fixator, if required due to the fracture morphology.

This study has certain limitations. Due to the retrospective character, follow-up was only possible in 116 patients after 6 weeks and 106 patients after 6 months. Due to the variety of fracture types and treatment options, proof of significant differences between these groups was difficult even with 121 cases. Also, the pain score was only evaluated on the third postoperative day and might be different at discharge or in follow-up.

## Conclusion

To our knowledge, this is the largest study evaluating the outcome after treatment of fragility fractures of the pelvis. This study shows that these challenging fractures can be treated safely and with good clinical outcome using navigated percutaneous SI or TSTI screws in combination with a supraacetabular external fixator if necessary. To increase stability and prevent loosening, TSTI screws should be preferred.

## Data Availability

All authors decided that the data and material will not be deposited in a public repository.
